# Bis{μ-(*E*)-methyl 4-[(2-carbamothio­ylhydrazinyl­idene)meth­yl]benzoate-κ^2^ *S*:*S*}bis­[iodido(triphenyl­phosphane-κ*P*)copper(I)]

**DOI:** 10.1107/S1600536811041845

**Published:** 2011-10-12

**Authors:** Soumik Mandal, Vamsidhar Nethi, Parna Gupta

**Affiliations:** a Department of Chemical Sciences, IISER Kolkata, Mohanpur Campus, 741 252 West Bengal, India

## Abstract

The title complex, [Cu_2_I_2_(C_10_H_11_N_3_O_2_S)_2_(C_18_H_15_P)_2_], is a centrosymmetric sulfur-bridged dimer of Cu^I^ with PPh_3_ and iodine. The Cu^I^ atom shows a distorted tetra­hedral geometry, with bite angles ranging from 98.61 (2) to 120.16 (3)°. The intra­molecular Cu⋯Cu distance is 2.8228 (12) Å. The thio­semicarbazone ligand is coordinated only through the S atom. In the crystal, the complex mol­ecules are linked *via* inter­molecular N—H⋯O hydrogen bonds, resulting in a hydrogen-bonded chain along the *b* axis.

## Related literature

For a related structure, see: Lobana *et al.* (2009 ▶). For the chemotherapeutic properties of transition metal complexes of thio­semicarbazones see: Quiroga *et al.* (1998 ▶). For binding modes of thio­semicarbazones, see: Dutta *et al.* (2008 ▶).
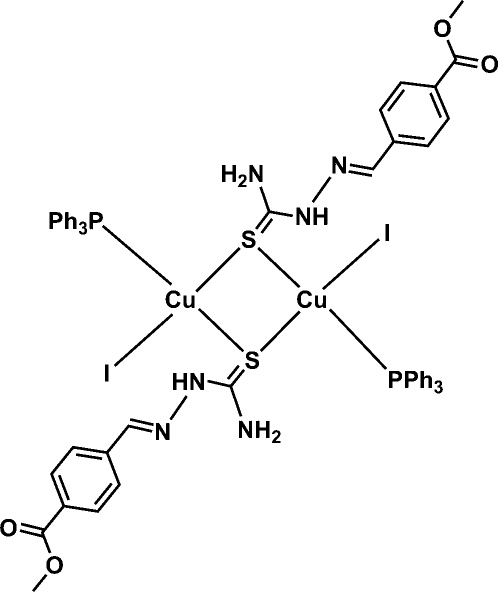


## Experimental

### 

#### Crystal data

[Cu_2_I_2_(C_10_H_11_N_3_O_2_S)_2_(C_18_H_15_P)_2_]
*M* *_r_* = 1380.02
Triclinic, 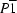

*a* = 9.6319 (16) Å
*b* = 11.945 (2) Å
*c* = 13.581 (4) Å
α = 108.627 (4)°
β = 101.655 (4)°
γ = 105.044 (3)°
*V* = 1359.0 (5) Å^3^
*Z* = 1
Mo *K*α radiation
μ = 2.11 mm^−1^
*T* = 100 K
0.18 × 0.11 × 0.09 mm

#### Data collection

Bruker APEXII CCD diffractometer
Absorption correction: multi-scan (*SADABS*; Bruker, 2008) ▶ *T*_min_ = 0.757, *T*_max_ = 0.827
22347 measured reflections
5922 independent reflections
4560 reflections with *I* > 2σ(*I*)
*R*_int_ = 0.065

#### Refinement

*R*[*F* ^2^ > 2σ(*F* ^2^)] = 0.036
*wR*(*F* ^2^) = 0.073
*S* = 1.02
5922 reflections
335 parameters
H-atom parameters constrained
Δρ_max_ = 1.56 e Å^−3^
Δρ_min_ = −0.82 e Å^−3^

### 

Data collection: *APEX2* (Bruker, 2008) ▶; cell refinement: *SAINT* (Bruker, 2008) ▶; data reduction: *SAINT* ▶; program(s) used to solve structure: *SHELXS97* (Sheldrick, 2008 ▶); program(s) used to refine structure: *SHELXL97* (Sheldrick, 2008 ▶); molecular graphics: *SHELXTL* (Sheldrick, 2008 ▶); software used to prepare material for publication: *SHELXTL*.

## Supplementary Material

Crystal structure: contains datablock(s) I, global. DOI: 10.1107/S1600536811041845/ds2140sup1.cif

Structure factors: contains datablock(s) I. DOI: 10.1107/S1600536811041845/ds2140Isup2.hkl

Additional supplementary materials:  crystallographic information; 3D view; checkCIF report

## Figures and Tables

**Table 1:** Hydrogen-bond geometry (Å, °)

*D*—H⋯*A*	*D*—H	H⋯*A*	*D*⋯*A*	*D*—H⋯*A*
N3—H3*B*⋯O1^i^	0.86	2.17	2.963 (5)	154

Symmetry code: (i) 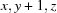.

**Fig. 1.**
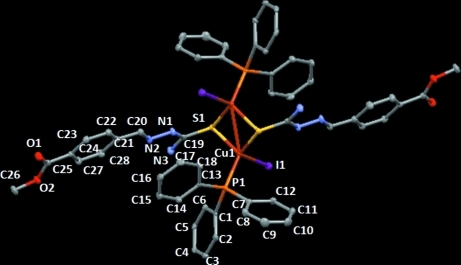
Structure with 50% probability displacement ellipsoids.

**Fig. 2.**
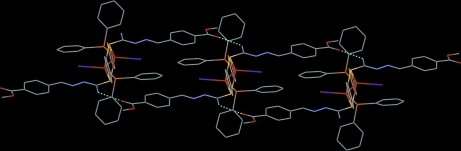
One dimensional asoociation via intermolecular H-bonding between C═O and N—H proton.

**Fig. 3.**
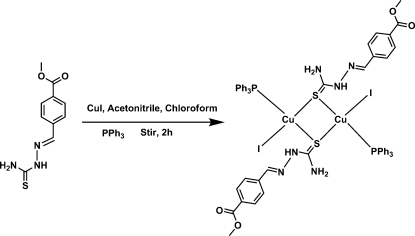
The formation of the title compound.

## supplementary crystallographic information

### Comment

Thiosemicarbazones and more specifically transition metal complexes of thiosemicarbazones (Lobana *et al.*, 2009) are of considerable pharmacological interest as they have shown a broad spectrum of chemotherapeutic properties (Quiroga *et al.*, 1998). Thiosemicarbazones usually bind to a metal ion in mono, bi or tridentate fashion (Dutta *et al.*, 2008). Interestingly, one-dimensional network formation has been observed here, where the single unit contain two metal ion bridged by the sulfur atom of two ligands. Two phosphine molecules and two iodine molecules are also coordinated to the metal centre. The C—S bond length [C19—S1 (1.720 (3) Å), C20—N2 (1.282 (5) Å)] indicates presence of double-bond character.

### Experimental

0.190 g(1.0 mmol) CuI was dissolved in 10 mL acetonitrile and 10 mL me thanol, and to this solution 0.237 g(1.0 mmol) Schiff's base of 4-formyl methyl benzoate and thiosemicarbazone was added and stirred for 2 h followed by the addition of 0.262 g(1.0 mmol) PPh_3_. The mixture was stirred for another 2 h, filtered and kept for crystallization. From the yellow solution yellow coloured block shaped crystals were obtained suitable for *x*-ray crystallography.

### Crystal data

**Table:**

[Cu_2_I_2_(C_10_H_11_N_3_O_2_S)_2_(C_18_H_15_P)_2_]	*Z* = 1
*M**_r_* = 1380.02	*F*(000) = 688
Triclinic, *P*1	*D*_x_ = 1.684 Mg m^−^^3^
Hall symbol: -P 1	Mo *K*α radiation, λ = 0.71073 Å
*a* = 9.6319 (16) Å	Cell parameters from 4585 reflections
*b* = 11.945 (2) Å	θ = 2.3–28.2°
*c* = 13.581 (4) Å	µ = 2.11 mm^−^^1^
α = 108.627 (4)°	*T* = 100 K
β = 101.655 (4)°	Block, yellow
γ = 105.044 (3)°	0.18 × 0.11 × 0.09 mm
*V* = 1359.0 (5) Å^3^	

### Data collection

**Table:**

Bruker APEXII CCD diffractometer	5922 independent reflections
Radiation source: fine-focus sealed tube	4560 reflections with *I* > 2σ(*I*)
graphite	*R*_int_ = 0.065
φ and ω scans	θ_max_ = 27.0°, θ_min_ = 1.7°
Absorption correction: multi-scan (*SADABS*; Bruker, 2008)	*h* = −12→11
*T*_min_ = 0.757, *T*_max_ = 0.827	*k* = −14→15
22347 measured reflections	*l* = −17→17

### Refinement

**Table:**

Refinement on *F*^2^	Primary atom site location: structure-invariant direct methods
Least-squares matrix: full	Secondary atom site location: difference Fourier map
*R*[*F*^2^ > 2σ(*F*^2^)] = 0.036	Hydrogen site location: inferred from neighbouring sites
*wR*(*F*^2^) = 0.073	H-atom parameters constrained
*S* = 1.02	*w* = 1/[σ^2^(*F*_o_^2^) + (0.0265*P*)^2^ + 0.8634*P*] where *P* = (*F*_o_^2^ + 2*F*_c_^2^)/3
5922 reflections	(Δ/σ)_max_ = 0.001
335 parameters	Δρ_max_ = 1.56 e Å^−^^3^
0 restraints	Δρ_min_ = −0.82 e Å^−^^3^

### Special details

**Table:**

Geometry. All e.s.d.'s (except the e.s.d. in the dihedral angle between two l.s. planes) are estimated using the full covariance matrix. The cell e.s.d.'s are taken into account individually in the estimation of e.s.d.'s in distances, angles and torsion angles; correlations between e.s.d.'s in cell parameters are only used when they are defined by crystal symmetry. An approximate (isotropic) treatment of cell e.s.d.'s is used for estimating e.s.d.'s involving l.s. planes.
Refinement. Refinement of *F*^2^ against ALL reflections. The weighted *R*-factor *wR* and goodness of fit *S* are based on *F*^2^, conventional *R*-factors *R* are based on *F*, with *F* set to zero for negative *F*^2^. The threshold expression of *F*^2^ > σ(*F*^2^) is used only for calculating *R*-factors(gt) *etc*. and is not relevant to the choice of reflections for refinement. *R*-factors based on *F*^2^ are statistically about twice as large as those based on *F*, and *R*- factors based on ALL data will be even larger.

### Fractional atomic coordinates and isotropic or equivalent isotropic displacement parameters (Å^2^)

**Table:**

	*x*	*y*	*z*	*U*_iso_*/*U*_eq_	
Cu1	0.44847 (4)	0.99151 (4)	0.39202 (4)	0.01460 (11)	
I1	0.44275 (2)	1.19639 (2)	0.35819 (2)	0.01695 (7)	
S1	0.70887 (9)	1.06875 (7)	0.50340 (8)	0.01341 (19)	
P1	0.36351 (9)	0.82308 (7)	0.23174 (8)	0.01134 (19)	
O1	0.9554 (3)	0.1581 (2)	0.2812 (2)	0.0209 (6)	
O2	1.0438 (3)	0.2624 (2)	0.1819 (2)	0.0216 (6)	
N1	0.7579 (3)	0.8525 (2)	0.4633 (3)	0.0153 (7)	
H1	0.7016	0.8385	0.5030	0.018*	
N2	0.8214 (3)	0.7653 (3)	0.4179 (3)	0.0178 (7)	
N3	0.8721 (3)	0.9774 (3)	0.3848 (3)	0.0190 (7)	
H3A	0.9110	0.9222	0.3577	0.023*	
H3B	0.8903	1.0452	0.3724	0.023*	
C1	0.4266 (3)	0.8438 (3)	0.1181 (3)	0.0124 (7)	
C2	0.3426 (4)	0.7719 (3)	0.0089 (3)	0.0158 (8)	
H2	0.2443	0.7175	−0.0092	0.019*	
C3	0.4045 (4)	0.7809 (3)	−0.0733 (3)	0.0187 (8)	
H3	0.3484	0.7315	−0.1462	0.022*	
C4	0.5499 (4)	0.8635 (3)	−0.0468 (3)	0.0177 (8)	
H4	0.5918	0.8688	−0.1018	0.021*	
C5	0.6322 (4)	0.9377 (3)	0.0606 (3)	0.0179 (8)	
H5	0.7291	0.9942	0.0776	0.021*	
C6	0.5724 (3)	0.9295 (3)	0.1446 (3)	0.0148 (8)	
H6	0.6287	0.9804	0.2171	0.018*	
C7	0.1577 (4)	0.7719 (3)	0.1779 (3)	0.0140 (7)	
C8	0.0617 (4)	0.6465 (3)	0.1235 (3)	0.0193 (8)	
H8	0.1016	0.5821	0.1127	0.023*	
C9	−0.0938 (4)	0.6175 (3)	0.0851 (3)	0.0244 (9)	
H9	−0.1572	0.5336	0.0494	0.029*	
C10	−0.1549 (4)	0.7110 (3)	0.0992 (4)	0.0242 (9)	
H10	−0.2590	0.6905	0.0733	0.029*	
C11	−0.0610 (4)	0.8364 (3)	0.1523 (3)	0.0223 (9)	
H11	−0.1018	0.9001	0.1607	0.027*	
C12	0.0937 (4)	0.8664 (3)	0.1926 (3)	0.0186 (8)	
H12	0.1560	0.9505	0.2300	0.022*	
C13	0.3993 (3)	0.6799 (3)	0.2321 (3)	0.0120 (7)	
C14	0.4589 (4)	0.6129 (3)	0.1569 (3)	0.0195 (8)	
H14	0.4803	0.6399	0.1028	0.023*	
C15	0.4858 (4)	0.5062 (3)	0.1631 (3)	0.0208 (9)	
H15	0.5253	0.4623	0.1129	0.025*	
C16	0.4549 (4)	0.4645 (3)	0.2425 (3)	0.0196 (8)	
H16	0.4725	0.3925	0.2454	0.023*	
C17	0.3975 (4)	0.5306 (3)	0.3180 (3)	0.0231 (9)	
H17	0.3759	0.5026	0.3716	0.028*	
C18	0.3722 (4)	0.6386 (3)	0.3138 (3)	0.0178 (8)	
H18	0.3367	0.6839	0.3662	0.021*	
C19	0.7841 (3)	0.9580 (3)	0.4455 (3)	0.0145 (8)	
C20	0.7924 (4)	0.6687 (3)	0.4428 (3)	0.0176 (8)	
H20	0.7346	0.6633	0.4894	0.021*	
C21	0.8490 (4)	0.5666 (3)	0.3989 (3)	0.0166 (8)	
C22	0.8040 (4)	0.4589 (3)	0.4212 (3)	0.0173 (8)	
H22	0.7422	0.4548	0.4654	0.021*	
C23	0.8497 (4)	0.3586 (3)	0.3787 (3)	0.0160 (8)	
H23	0.8207	0.2882	0.3955	0.019*	
C24	0.9396 (4)	0.3629 (3)	0.3104 (3)	0.0142 (8)	
C25	0.9797 (4)	0.2505 (3)	0.2592 (3)	0.0157 (8)	
C26	1.0860 (4)	0.1589 (3)	0.1253 (4)	0.0257 (9)	
H26A	1.1516	0.1418	0.1774	0.038*	
H26B	1.1378	0.1804	0.0768	0.038*	
H26C	0.9969	0.0855	0.0838	0.038*	
C27	0.9853 (4)	0.4709 (3)	0.2880 (3)	0.0197 (8)	
H27	1.0460	0.4748	0.2430	0.024*	
C28	0.9406 (4)	0.5719 (3)	0.3325 (3)	0.0206 (8)	
H28	0.9721	0.6437	0.3179	0.025*	

### Atomic displacement parameters (Å^2^)

**Table:**

	*U*^11^	*U*^22^	*U*^33^	*U*^12^	*U*^13^	*U*^23^
Cu1	0.0178 (2)	0.01218 (19)	0.0124 (3)	0.00618 (16)	0.00386 (19)	0.00278 (18)
I1	0.02130 (12)	0.01412 (11)	0.01860 (16)	0.00695 (8)	0.00813 (10)	0.00863 (10)
S1	0.0140 (4)	0.0123 (4)	0.0132 (5)	0.0046 (3)	0.0041 (4)	0.0041 (3)
P1	0.0139 (4)	0.0109 (4)	0.0100 (5)	0.0060 (3)	0.0040 (4)	0.0036 (4)
O1	0.0301 (13)	0.0156 (12)	0.0252 (17)	0.0118 (10)	0.0147 (13)	0.0116 (12)
O2	0.0278 (13)	0.0214 (12)	0.0221 (17)	0.0139 (10)	0.0150 (13)	0.0080 (12)
N1	0.0185 (14)	0.0125 (13)	0.0210 (19)	0.0086 (11)	0.0123 (14)	0.0077 (13)
N2	0.0154 (13)	0.0147 (13)	0.023 (2)	0.0077 (11)	0.0086 (14)	0.0040 (13)
N3	0.0211 (15)	0.0206 (14)	0.024 (2)	0.0107 (12)	0.0140 (15)	0.0131 (14)
C1	0.0162 (15)	0.0109 (14)	0.015 (2)	0.0098 (12)	0.0073 (15)	0.0064 (14)
C2	0.0168 (16)	0.0126 (15)	0.017 (2)	0.0072 (13)	0.0058 (16)	0.0022 (15)
C3	0.0261 (18)	0.0211 (17)	0.012 (2)	0.0142 (14)	0.0053 (16)	0.0058 (16)
C4	0.0234 (17)	0.0262 (18)	0.018 (2)	0.0180 (15)	0.0133 (17)	0.0153 (17)
C5	0.0124 (15)	0.0228 (17)	0.027 (2)	0.0104 (13)	0.0082 (16)	0.0164 (17)
C6	0.0141 (15)	0.0163 (16)	0.016 (2)	0.0077 (13)	0.0043 (15)	0.0072 (15)
C7	0.0152 (16)	0.0154 (15)	0.010 (2)	0.0037 (12)	0.0035 (15)	0.0049 (14)
C8	0.0184 (17)	0.0156 (16)	0.024 (2)	0.0059 (13)	0.0089 (16)	0.0060 (16)
C9	0.0186 (17)	0.0183 (17)	0.030 (3)	−0.0011 (14)	0.0093 (18)	0.0066 (17)
C10	0.0131 (16)	0.031 (2)	0.028 (3)	0.0070 (14)	0.0061 (17)	0.0117 (18)
C11	0.0237 (18)	0.0280 (19)	0.022 (2)	0.0162 (15)	0.0084 (17)	0.0125 (18)
C12	0.0208 (17)	0.0131 (15)	0.017 (2)	0.0058 (13)	0.0029 (16)	0.0025 (15)
C13	0.0118 (15)	0.0122 (14)	0.009 (2)	0.0041 (12)	0.0017 (14)	0.0022 (14)
C14	0.0264 (18)	0.0204 (17)	0.017 (2)	0.0119 (15)	0.0106 (17)	0.0078 (16)
C15	0.0296 (19)	0.0184 (17)	0.020 (2)	0.0161 (15)	0.0096 (18)	0.0070 (16)
C16	0.0229 (17)	0.0137 (16)	0.022 (2)	0.0090 (14)	0.0035 (17)	0.0068 (16)
C17	0.033 (2)	0.0217 (18)	0.020 (2)	0.0105 (16)	0.0099 (19)	0.0133 (17)
C18	0.0258 (18)	0.0164 (16)	0.017 (2)	0.0115 (14)	0.0135 (17)	0.0069 (16)
C19	0.0113 (15)	0.0138 (15)	0.013 (2)	0.0022 (12)	0.0008 (14)	0.0015 (14)
C20	0.0157 (16)	0.0180 (16)	0.019 (2)	0.0063 (13)	0.0082 (16)	0.0057 (16)
C21	0.0165 (16)	0.0161 (16)	0.016 (2)	0.0051 (13)	0.0051 (16)	0.0047 (15)
C22	0.0147 (16)	0.0200 (17)	0.018 (2)	0.0057 (13)	0.0077 (15)	0.0068 (16)
C23	0.0142 (15)	0.0145 (15)	0.020 (2)	0.0033 (13)	0.0061 (16)	0.0090 (15)
C24	0.0143 (15)	0.0153 (15)	0.014 (2)	0.0055 (12)	0.0046 (15)	0.0060 (15)
C25	0.0146 (16)	0.0139 (15)	0.015 (2)	0.0031 (12)	0.0029 (15)	0.0034 (15)
C26	0.032 (2)	0.0228 (18)	0.028 (3)	0.0159 (16)	0.0186 (19)	0.0066 (18)
C27	0.0217 (17)	0.0209 (17)	0.024 (2)	0.0107 (14)	0.0132 (17)	0.0115 (17)
C28	0.0291 (19)	0.0164 (16)	0.023 (2)	0.0106 (14)	0.0127 (18)	0.0114 (16)

### Geometric parameters (Å, °)

**Table:**

Cu1—P1	2.2548 (10)	C9—C10	1.371 (5)
Cu1—S1^i^	2.4060 (10)	C9—H9	0.9300
Cu1—S1	2.4093 (10)	C10—C11	1.387 (5)
Cu1—I1	2.6423 (6)	C10—H10	0.9300
Cu1—Cu1^i^	2.8228 (12)	C11—C12	1.384 (5)
S1—C19	1.720 (3)	C11—H11	0.9300
S1—Cu1^i^	2.4059 (10)	C12—H12	0.9300
P1—C7	1.827 (3)	C13—C18	1.394 (5)
P1—C1	1.832 (4)	C13—C14	1.402 (5)
P1—C13	1.832 (3)	C14—C15	1.389 (5)
O1—C25	1.212 (4)	C14—H14	0.9300
O2—C25	1.347 (4)	C15—C16	1.377 (6)
O2—C26	1.437 (4)	C15—H15	0.9300
N1—C19	1.329 (4)	C16—C17	1.385 (5)
N1—N2	1.383 (4)	C16—H16	0.9300
N1—H1	0.8600	C17—C18	1.390 (5)
N2—C20	1.282 (5)	C17—H17	0.9300
N3—C19	1.325 (5)	C18—H18	0.9300
N3—H3A	0.8600	C20—C21	1.467 (5)
N3—H3B	0.8600	C20—H20	0.9300
C1—C2	1.390 (5)	C21—C28	1.387 (5)
C1—C6	1.402 (4)	C21—C22	1.395 (5)
C2—C3	1.388 (5)	C22—C23	1.377 (5)
C2—H2	0.9300	C22—H22	0.9300
C3—C4	1.384 (5)	C23—C24	1.394 (5)
C3—H3	0.9300	C23—H23	0.9300
C4—C5	1.372 (5)	C24—C27	1.400 (5)
C4—H4	0.9300	C24—C25	1.489 (5)
C5—C6	1.396 (5)	C26—H26A	0.9600
C5—H5	0.9300	C26—H26B	0.9600
C6—H6	0.9300	C26—H26C	0.9600
C7—C8	1.394 (4)	C27—C28	1.384 (5)
C7—C12	1.401 (5)	C27—H27	0.9300
C8—C9	1.391 (5)	C28—H28	0.9300
C8—H8	0.9300		
			
P1—Cu1—S1^i^	104.59 (4)	C12—C11—C10	119.8 (3)
P1—Cu1—S1	120.16 (3)	C12—C11—H11	120.1
S1^i^—Cu1—S1	108.22 (3)	C10—C11—H11	120.1
P1—Cu1—I1	110.45 (3)	C11—C12—C7	120.9 (3)
S1^i^—Cu1—I1	115.32 (3)	C11—C12—H12	119.5
S1—Cu1—I1	98.61 (2)	C7—C12—H12	119.5
P1—Cu1—Cu1^i^	130.06 (4)	C18—C13—C14	118.4 (3)
S1^i^—Cu1—Cu1^i^	54.16 (3)	C18—C13—P1	118.5 (3)
S1—Cu1—Cu1^i^	54.05 (3)	C14—C13—P1	123.0 (3)
I1—Cu1—Cu1^i^	119.49 (2)	C15—C14—C13	120.1 (4)
C19—S1—Cu1^i^	113.92 (13)	C15—C14—H14	120.0
C19—S1—Cu1	106.03 (11)	C13—C14—H14	120.0
Cu1^i^—S1—Cu1	71.78 (3)	C16—C15—C14	121.0 (4)
C7—P1—C1	103.42 (17)	C16—C15—H15	119.5
C7—P1—C13	104.04 (14)	C14—C15—H15	119.5
C1—P1—C13	102.23 (15)	C15—C16—C17	119.5 (3)
C7—P1—Cu1	109.73 (12)	C15—C16—H16	120.2
C1—P1—Cu1	117.80 (11)	C17—C16—H16	120.2
C13—P1—Cu1	117.87 (12)	C16—C17—C18	120.1 (4)
C25—O2—C26	116.7 (3)	C16—C17—H17	120.0
C19—N1—N2	119.7 (3)	C18—C17—H17	120.0
C19—N1—H1	120.1	C17—C18—C13	120.9 (3)
N2—N1—H1	120.1	C17—C18—H18	119.5
C20—N2—N1	114.6 (3)	C13—C18—H18	119.5
C19—N3—H3A	120.0	N3—C19—N1	118.8 (3)
C19—N3—H3B	120.0	N3—C19—S1	120.9 (3)
H3A—N3—H3B	120.0	N1—C19—S1	120.3 (3)
C2—C1—C6	119.4 (3)	N2—C20—C21	121.1 (3)
C2—C1—P1	123.3 (2)	N2—C20—H20	119.5
C6—C1—P1	117.1 (3)	C21—C20—H20	119.5
C3—C2—C1	120.5 (3)	C28—C21—C22	119.2 (3)
C3—C2—H2	119.8	C28—C21—C20	122.0 (3)
C1—C2—H2	119.8	C22—C21—C20	118.8 (3)
C4—C3—C2	119.9 (4)	C23—C22—C21	121.0 (3)
C4—C3—H3	120.0	C23—C22—H22	119.5
C2—C3—H3	120.0	C21—C22—H22	119.5
C5—C4—C3	120.1 (4)	C22—C23—C24	119.9 (3)
C5—C4—H4	119.9	C22—C23—H23	120.1
C3—C4—H4	119.9	C24—C23—H23	120.1
C4—C5—C6	120.8 (3)	C23—C24—C27	119.3 (3)
C4—C5—H5	119.6	C23—C24—C25	119.3 (3)
C6—C5—H5	119.6	C27—C24—C25	121.4 (3)
C5—C6—C1	119.2 (3)	O1—C25—O2	123.5 (3)
C5—C6—H6	120.4	O1—C25—C24	125.2 (3)
C1—C6—H6	120.4	O2—C25—C24	111.3 (3)
C8—C7—C12	118.4 (3)	O2—C26—H26A	109.5
C8—C7—P1	124.6 (3)	O2—C26—H26B	109.5
C12—C7—P1	117.0 (2)	H26A—C26—H26B	109.5
C9—C8—C7	120.1 (3)	O2—C26—H26C	109.5
C9—C8—H8	120.0	H26A—C26—H26C	109.5
C7—C8—H8	120.0	H26B—C26—H26C	109.5
C10—C9—C8	120.9 (3)	C28—C27—C24	120.3 (4)
C10—C9—H9	119.6	C28—C27—H27	119.8
C8—C9—H9	119.6	C24—C27—H27	119.8
C9—C10—C11	119.9 (3)	C27—C28—C21	120.3 (3)
C9—C10—H10	120.1	C27—C28—H28	119.9
C11—C10—H10	120.1	C21—C28—H28	119.9

Symmetry codes: (i) −*x*+1, −*y*+2, −*z*+1.

### Hydrogen-bond geometry (Å, °)

**Table:**

*D*—H···*A*	*D*—H	H···*A*	*D*···*A*	*D*—H···*A*
N3—H3B···O1^ii^	0.86	2.17	2.963 (5)	154.

Symmetry codes: (ii) *x*, *y*+1, *z*.
